# Thermal Treatment and Polymer Matrix Diffusion Effects on Hydroxyapatite Particle Size Evolution

**DOI:** 10.3390/polym17172323

**Published:** 2025-08-27

**Authors:** Alexandru Pahomi, Ionela-Amalia Potinteu, Alexandra-Maria Tășală, Bianca-Denisa Cernușcă, Paula Sfîrloagă, Titus Vlase, Gabriela Vlase, Mihaela Maria Budiul

**Affiliations:** 1 Research Centre for Thermal Analysis in Environmental Problems, West University of Timisoara, Pestalozzi Street 16, 300115 Timisoara, Romania; alexandru.pahomi@e-uvt.ro (A.P.); ionela.potinteu@e-uvt.ro (I.-A.P.); alexandra.tasala@e-uvt.ro (A.-M.T.); bianca.cernusca@e-uvt.ro (B.-D.C.); titus.vlase@e-uvt.ro (T.V.); 2 ICAM—Advanced Environmental Research Institute, West University of Timisoara, Oituz Street 4, 300086 Timisoara, Romania; 3 National Institute for Research and Development in Electrochemistry and Condensed Matter, Dr. A. P. Podeanu Street 144, 300569 Timisoara, Romania; paulasfirloaga@gmail.com; 4 Doctoral School of Exact Sciences and Natural Sciences, West University of Timisoara, Pestalozzi Street 16, 300115 Timisoara, Romania

**Keywords:** thermal treatment, hydroxyapatite, chitosan, sodium alginate, polymers

## Abstract

A widely used approach for synthesizing hydroxyapatite (HA) particles is the wet chemical precipitation method, favoured for its cost-effectiveness and straightforward process. Incorporating organic macromolecules with polar functional groups, such as COOH and OH, during synthesis can impact the properties of the resulting HA particles. These functional groups enhance the affinity for positively charged Ca^2+^ ions, promoting HA crystal nucleation in the solution. In this study, solutions at different concentrations of chitosan and sodium alginate are used as nucleation medium for the HA particles in order to decrease their particle size. The calcium and phosphate precursor solutions were adjusted to a pH of 12 and added to the polymer solution with a concentration varying from 5 to 10% *w*/*v*, reported to the stoichiometric mass of HA according to the synthesis reaction. After synthesis, the resulting powder was calcinated at 1000 °C. The effects that the polymers have on the properties of HA particles were monitored using SEM, FT-IR, EDAX, DLS, and TGA before and after the thermal treatment to see how the system evolves till crystallization of HA occurs. The largest decrease in average particle diameter—67.7%—was observed in the HA + Alg 10% sample, although a reduction in particle size was evident in all samples.

## 1. Introduction

A popular method for synthesizing hydroxyapatite (HA) is the chemical precipitation method, which is favoured due to its low reagent cost and simplicity of the required steps [1]. By using macromolecules with functional groups during the synthesis—groups such as -COOH and -OH—a change in the properties of the synthesized particles was observed. The functional groups have a higher affinity for Ca^2+^ cations and facilitate the nucleation of HA crystals in solution [2,3].

The particle size plays a crucial role in determining morphological properties and affects the quality of fillers used as reinforcing agents [4]. Smaller particles have a larger contact area per unit weight than larger particles, which increases the contact between the polymer and the HA particles, which can lead to better dispersion of the particles in the polymer matrix and avoid agglomerate formation [5,6,7,8,9]. Larger particles can also act as points of localized stress and create defects in the composite material, which can lead to material failure at low deformation. However, the use of nanoparticles is associated with significant disadvantages, including the complexity and high cost of their production compared to macro- and microparticles [10,11,12]. Hydroxyapatite with a small particle size provides a higher surface area-to-volume ratio, enabling stronger interaction with the surrounding tissues, while also blending more uniformly with polymeric or collagen matrices to yield mechanically robust and flexible composites [13].

Chitosan (Chit) is a versatile natural polymer obtained from the partial deacetylation of chitin under alkaline conditions and is a copolymer of glucosamine and N-acetylglucosamine, forming a linear chain linked by β-1→4 bonds [14]. Conjugation between chitosan and HA occurs through the interaction of the calcium ions of HA with the amino groups of chitosan, which serve as nucleation sites for the growth of HA crystals and make them mechanically flexible [15].

Similarly to chitosan, alginate (Alg) is a polymer of marine origin obtained from brown algae. Glucuronic acid and mannuronic acid copolymerise via α-1,4-glycosidic bonds and form alginates [12,16,17]. The Ca^2+^-containing precursors from HA synthesis bind to the carboxyl groups of the sodium alginates via strong electrostatic bonds. The addition of phosphate precursors to this calcium–alginate complex causes the PO_4_^3−^ ions to interact with the calcium in the complex, which leads to supersaturation and thus to the nucleation of hydroxyapatite [5,11].

In this study, solutions with different concentrations of chitosan and sodium alginate are used as nucleation media for HA particles to obtain smaller-size particles. This synthesis process supports the wet precipitation synthesis method, a method in which the mean size of the particles cannot be controlled [18,19]. The calcium and phosphate precursor solutions are first adjusted to a pH of 12 and added to the polymer solution at concentrations of 5% and 10% (*w*/*v*), respectively, based on the stoichiometric mass of HA according to the synthesis reaction. After synthesis, part of the resulting powder is calcined at 1000 °C to ensure that any non-stoichiometric hydroxyapatite decomposes into β-TCP, which typically forms alongside hydroxyapatite, or other calcium phosphates [20]. The properties of the initially obtained and the calcined particles are compared, using both the original synthesis product and the product obtained after dispersion in the polymer matrix.

The effects of the polymers on the physicochemical properties of the HA particles are determined using analytical techniques such as SEM, FT-IR, EDAX, DLS, XRD, and TG before and after thermal treatment, in order to follow the evolution of the properties.

## 2. Materials and Methods

The substances used for the synthesis of hydroxyapatite are Ca(NO_3_)_2_∙4H_2_O (KEBO Lab, Neuhausen, Switzerland), NH_4_H_2_PO_4_ (Sigma Aldrich, Saint Louis, MO, USA), and 25% aqueous NH_3_ (Sigma Aldrich). The substances used to prepare the polymer solutions are glacial acetic acid (Sigma Aldrich), low-viscosity chitosan (Sigma Aldrich), and sodium alginate (VWR Prolabo, Randor, PA, USA). The reference for the synthesized hydroxyapatite is 5 µm hydroxyapatite purchased from Sigma Aldrich. All reagents used are of analytical purity, and distilled water is used to prepare the solutions.

To synthesize hydroxyapatite, Ca(NO_3_)_2_∙4H_2_O is dissolved in distilled water and added dropwise to the solution containing the phosphorus precursor while stirring. The concentration of the solutions is calculated so that the Ca/P ratio is 1.667, and the pH value of each solution is adjusted to 12 beforehand with ammonia.

10Ca(NO_3_)_2_ + 6NH_4_H_2_PO_4_ + 14NH_4_OH→Ca_10_(PO_4_)_6_(OH)_2_ + 20NH_4_NO_3_ + 12H_2_O

The pH of the mixture is adjusted to 12 and stirred magnetically for 2 h, monitoring the pH and adjusting it if necessary. If the pH value is kept at 12, the formation of HA is ensured. At lower pH values, other calcium phosphate phases might precipitate instead of or alongside HA [21,22]. After the 2 h of stirring, the mixture is left to stand for 24 h to allow the precipitate to mature and reach equilibrium.

The method for preparing the polymer solutions is adapted from the protocols used by Tsiourvas, Li, and their co-authors [23,24]. Low-viscosity chitosan (Chit) is dissolved in a 1% (*v*/*v*) aqueous acetic acid solution to obtain a homogeneous chitosan solution of 5% and 10% (*w*/*v*) based on the experimentally calculated mass of HA. Sodium alginate (Alg) is dissolved in distilled water until a homogeneous sodium alginate solution of 5% and 10% (*w*/*v*), based on the experimentally calculated mass of HA, is obtained. The concentrations of the polymer solutions relative to the mass of the stoichiometrically obtained hydroxyapatite are 0.5 and 1% (*w*/*v*) for both polymers and were chosen to ensure a moderate viscosity of the reaction medium. After complete dissolution of the polymers, the polymer solutions are completely added to the solution obtained in the hydroxyapatite synthesis containing the HA precursor, stirred at room temperature for 6 h, and sonicated for 30 min.

After the ultrasonic treatment, the solutions are vacuum filtered, and the product obtained is dried at 120 °C. To obtain hydroxyapatite in crystalline form and to volatilise the polymer component, the product obtained after drying is ground and calcined at 1000 °C for 12 h [25].

After the applied procedures, two sets of samples are obtained, which are summarized in Table 1.

The products synthesized were dried at 120 °C and analyzed in order to obtain an indication of how the thermal treatment affects certain properties.

The ATR-FT-IR analysis was performed with a Perkin-Elmer Spotlight 400+ Spectrum 100 FT-IR spectrometer (PerkinElmer Inc., Waltham, MA, USA) employing the Attenuated Total Reflectance (ATR) technique. The spectra were recorded in the range 4000–600 cm^−1^ at a resolution of 2 cm^−1^ after 10 recordings. A PANalytical diffractometer (Malvern Panalytical Ltd., Malvern, United Kingdom) with a Cu Kα radiation source (λ = 0.15406 nm) in the 2θ range from 10° to 80° was used for X-ray analysis. Thermal stability was determined using a Mettler Toledo TGA/DSC 1 analyser (Mettler-Toledo LLC, Columbus, OH, USA) in the temperature range of 30–900 °C at a heating rate of 10 °C/min in a nitrogen atmosphere (50 mL/min). The average particle size was measured by dynamic light scattering technique (DLS) using a Malvern Zetasizer Nano ZS (Malvern Panalytical Ltd., Malvern, United Kingdom)equipped with a He-Ne laser (633 nm) as an incident light source. Scanning electron microscopy (SEM) was performed with a JEOL JSM-IT 200 microscope ((JEOL Ltd., Tokyo, Japan) at an acceleration current of 10 kV in a low vacuum (30 Pa). The working distance was 10–11 mm, and the BED-S detector of the device was used. Energy dispersive X-ray spectroscopy (EDAX) was performed with a JEOL JSM IT-200 electron microscope in low vacuum (JEOL Ltd., Tokyo, Japan).

## 3. Results and Discussion

The first step was the characterization of the obtained products using FT-IR spectroscopy, thermal analysis, XRD, and EDAX. Then, using XRD, DLS, and SEM techniques, the dimensional parameters obtained were presented, and the influence of heat treatment on the modification of particle size was highlighted.

### 3.1. Sample Characterization

#### 3.1.1. ATR-FT-IR Analysis

**Figure 1:**
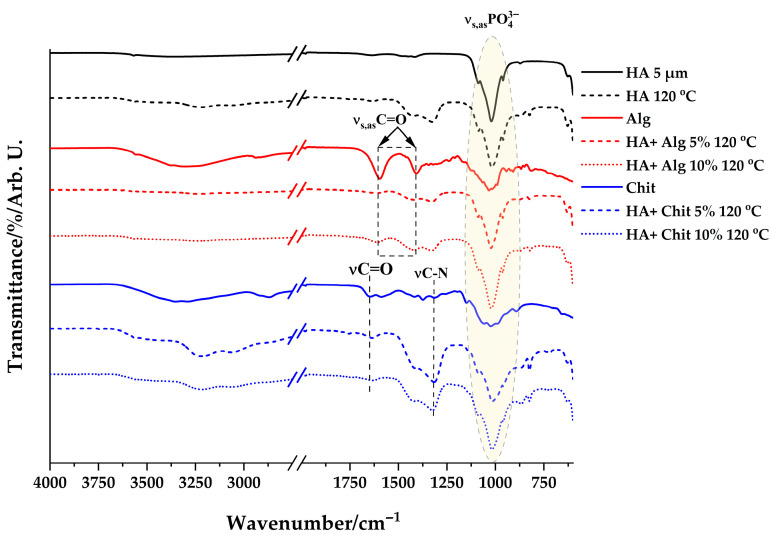
FT-IR spectra of samples dried at 120 °C.

**Figure 2:**
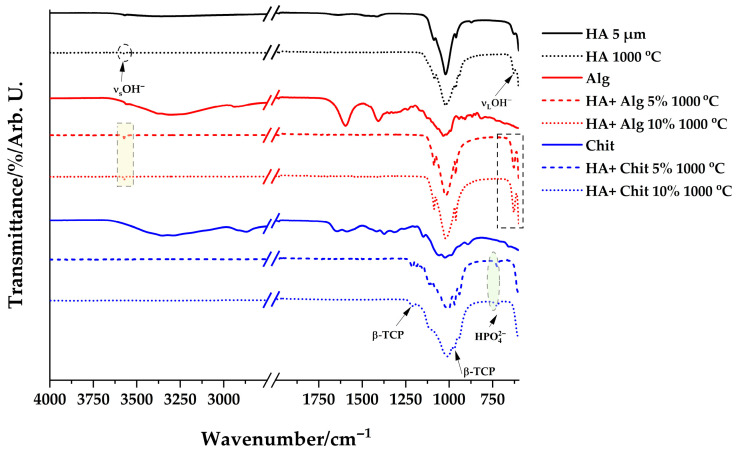
FT-IR spectra of samples dried at 1000 °C.

The FT-IR spectra of the obtained samples, dried and calcined at 1000 °C, are measured in the range 4000–600 cm^−1^ and are represented in Figure 1 and Figure 2. For reference, the spectra of the pure substances were also plotted.

In the case of hydroxyapatite, the bands present at 960 cm^−1^, 1025 cm^−1^ and 1090 cm^−1^ are attributed to the symmetric and asymmetric valence vibration of the PO_4_^3−^ group present in the hydroxyapatite structure and are observed in both spectra, both the spectrum of the pure substance and the synthesized hydroxyapatite. In the case of synthesized hydroxyapatite, the bands observed at 3573 cm^−1^ and 633 cm^−1^ are attributed to the symmetric and lattice valence vibration of the HO^−^ group. In addition, the two bands located in the spectrum of synthesized hydroxyapatite, at 870 and 1454 cm^−1^, are attributed to the CO_3_^2−^ group, due to the absorption of CO_2_ from the atmosphere during the synthesis. At 1332 and 824 cm^−1^, the observed bands correspond to the NO_3_^−^ group.

The FT-IR spectrum of pure alginate shows a wide absorption band in the range of 3600–3000 cm^−1^ due to the stretching vibration of the HO^−^ group and the vibration band at 2941 cm^−1^ of the C-H bond. The intense bands observed at 1593 cm^−1^ and 1410 cm^−1^ are attributed to the symmetric and asymmetric stretching of the C=O groups of –COO^−^, indicating the presence of the carboxylic acid group in the alginate structure. The bands appearing at 1068 and 1033 cm^−1^ are attributed to the stretching vibrations of the C-O bond and the stretching of the C-C bond in the pyranose rings.

In the case of pure chitosan, a strong band at 3360 cm^−1^ is observed together with a band at 3280 cm^−1^, which correspond to the stretching vibration of HO^−^ and the N-H bond, also attributed to intramolecular hydrogen bonds. The absorption bands at 2921 and 2877 cm^−1^ are attributed to the symmetric and asymmetric stretching vibration of the C-H bond. These bands are characteristic of polysaccharides [26,27]. The presence of residual N-acetyl groups is confirmed by the bands at 1646 cm^−1^ (stretching vibration of the C=O bond, amide band I) and 1325 cm^−1^ (stretching of the C-N bond, amide band III). The valence vibration of the CH_2_ groups and the asymmetric deformation of the CH_3_ groups are confirmed by the presence of the bands at 1420 and 1376 cm^−1^.

The absorption band found at 1152 cm^−1^ is attributed to the asymmetric stretching of the C-O-C bridge, and the bands at 1066 and 1028 cm^−1^ correspond to the stretching vibration of the C-O bond. The band at 896 cm^−1^ corresponds to the out-of-plane deformation of the C-H bond present in the monosaccharide ring.

It is observed that the presence of hydroxyapatite in polymer solutions leads to interactions between the two substances. First, it is observed in the spectra represented that the characteristic bands of hydroxyapatite are predominant, while those characteristic of polymers are covered. Secondly, the decrease in intensity of the band at 1593 cm^−1^, characteristic of alginate, and the disappearance of the band at 1325 cm^−1^, characteristic of chitosan, are observed.

After calcination, the complete disappearance of the bands characteristic of polymers is observed in all analyzed samples. Also, in the samples containing alginate, the bands characteristic of the HO^−^ group and the phosphate group are well highlighted. These bands appear in the spectrum of crystalline hydroxyapatite.

In contrast, in the spectra of samples containing chitosan, the bands characteristic of crystalline hydroxyapatite are not observed, but three other bands appear at 1222, 970, and 728 cm^−1^ in addition to those characteristic of the phosphate group. These bands indicate the formation of other phosphates following the calcination process, possibly β-tricalcium phosphate and hydrogen phosphate [28]. The band at 970 cm^−1^ is also distinguished in the case of calcined synthesized hydroxyapatite, which indicates the presence of another compound after calcination instead of hydroxyapatite.

#### 3.1.2. XRD Analysis

Following the observations made by FT-IR spectroscopy, X-ray diffraction (XRD) was used to identify the component phases in the synthesis products obtained and validate the production of hydroxyapatite.

**Figure 3:**
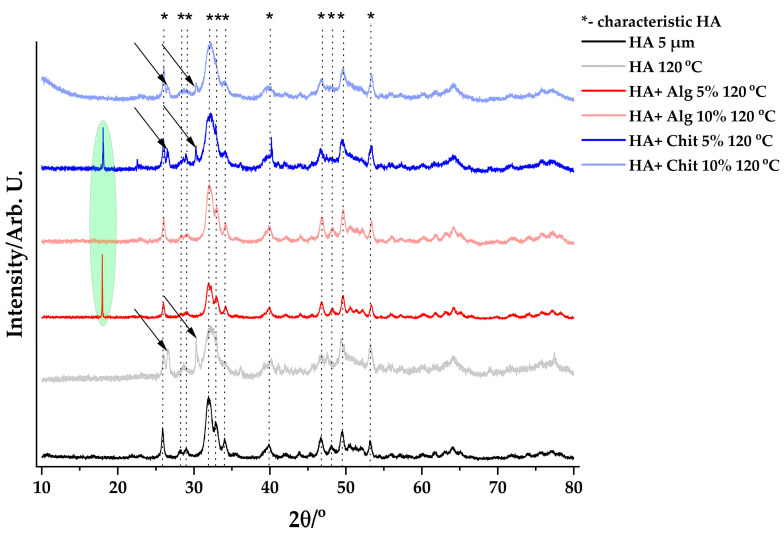
Representation of XRD peaks of products obtained by drying at 120 °C.

Figure 3 shows the diffraction peaks of samples dried at 120 °C, compared to standard hydroxyapatite.

Characteristic peaks of hydroxyapatite are distinguished in all samples, but the shape of the peaks is not very sharp. This indicates the formation of a hydroxyapatite with low crystallinity. In addition to these peaks, the presence of other peaks is observed. This is normal and due to the presence of unconsumed reagents in the samples [29,30,31].

**Figure 4:**
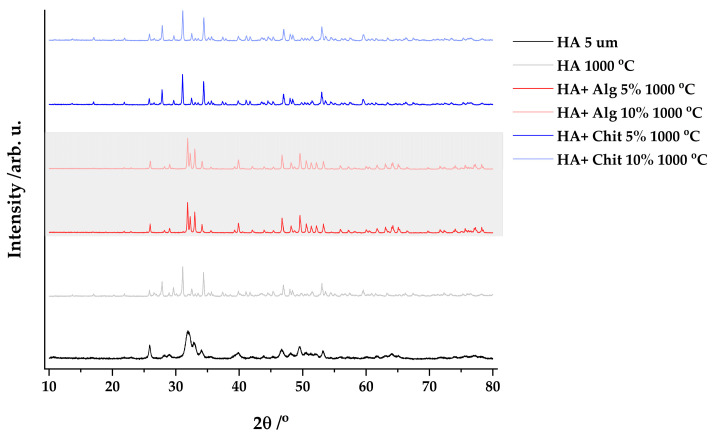
Representation of XRD peaks of products obtained by calcination at 1000 °C.

In Figure 4, a completely different shape of the peaks is observed after the heat treatment, and the observed peaks are used to identify the phases found in the calcined product. It is visible that the characteristic intensities of hydroxyapatite are fully found in the representation of the peaks of the samples containing alginate.

**Figure A1:**
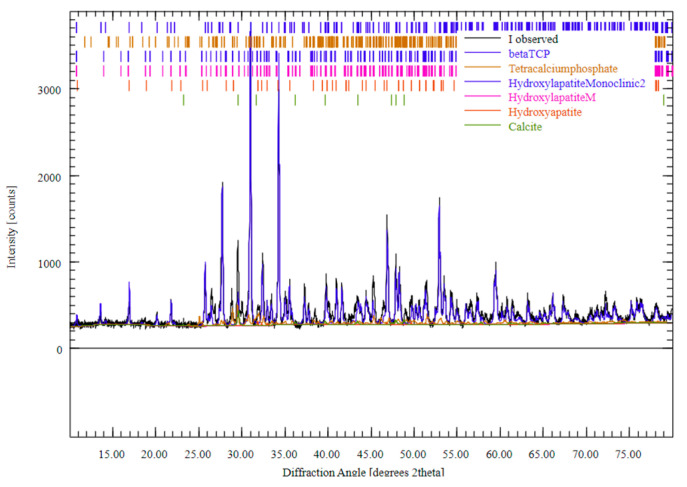
Rietveld method applied for the sample HA 1000 °C.

**Figure A2:**
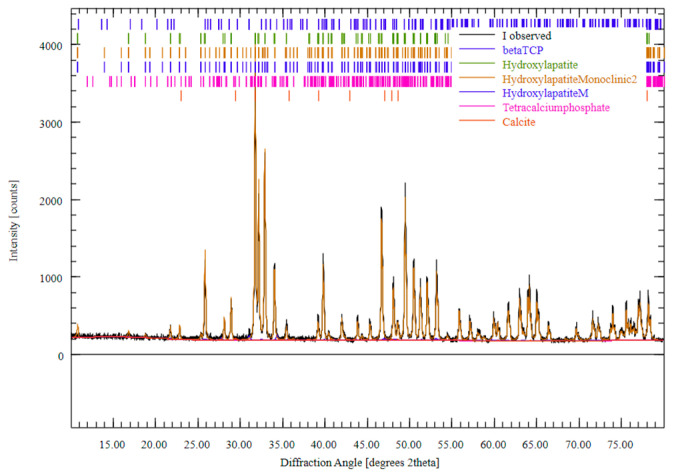
Rietveld method applied for the sample HA + Alg 5% 1000 °C.

**Figure A3:**
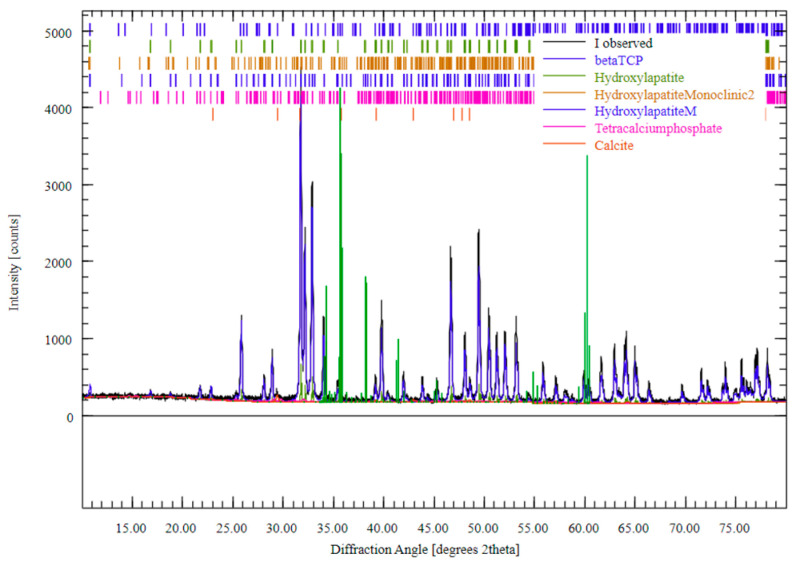
Rietveld method applied for the sample HA + Alg 10% 1000 °C.

**Figure A4:**
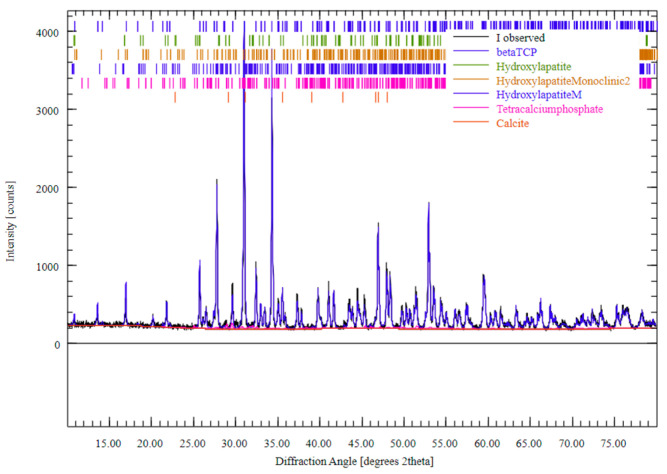
Rietveld method applied for the sample HA + Chit 5% 1000 °C.

**Figure A5:**
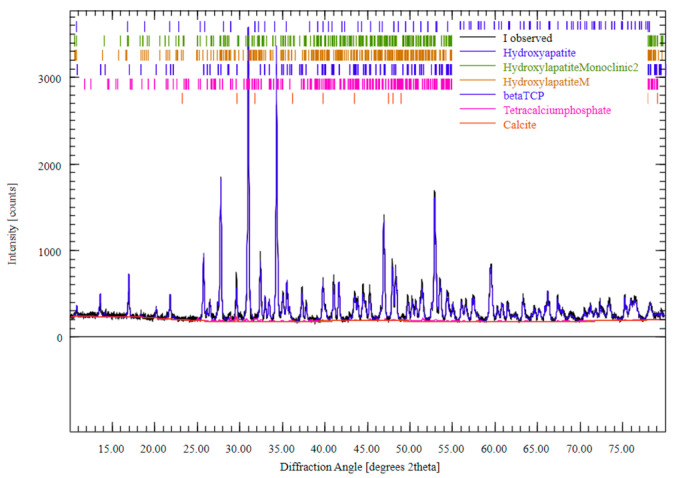
Rietveld method applied for the sample HA + Chit 10% 1000 °C.

Using data processing by the Rietveld method, the obtained diffraction peaks are compared with the Profex programme database, version 4.3.2a, and the composition of each sample is determined (see Appendix A, Figure A1, Figure A2, Figure A3, Figure A4 and Figure A5).

The phases identified, in different proportions, are hydroxyapatite, β-tricalcium phosphate, calcite (CaCO_3_), and tetracalcium phosphate (Ca_4_(PO_4_)_2_O). The percentage composition for each sample is summarized in the following Table 2.

After XRD analysis, the following observations are made:

- Samples containing alginate lead to the formation of a larger amount of hydroxyapatite after calcination.
- Calcite and tetracalcium phosphate are present in relatively small amounts in the calcined sample.
- The presence of chitosan leads to the formation of β-tricalcium phosphate—the predominant phase in the sample.
- The hydroxyapatite precursor is transformed, following heat treatment into β-tricalcium phosphate in a fairly high ratio for samples containing chitosan.
- For the HA + Alg 10% 1000 °C sample, the phases could not be fully identified, leaving 3.55% unassigned to a phase.

The high amount of β-tricalcium phosphate obtained in the chitosan-containing samples can be explained by the pH at which the reaction occurs. Chitosan is prepared in acetic acid, pH approximately 4, and this leads to a decrease in the pH of the mixture below 12 [32]. On the other hand, the presence of alginate in the solution has a beneficial effect, and due to the interaction between the polymer chain and Ca^2+^ ions, a better dispersion of the reagents in the solution is obtained.

The presence of secondary phases alongside hydroxyapatite can be beneficial for the use of the obtained materials in bone substitutes because they influence the material’s biocompatibility, bioactivity, mechanical properties, and degradation rate. These phases contribute to a more natural bone-like structure and can promote bone regeneration more effectively than pure hydroxyapatite [33,34,35].

#### 3.1.3. Thermal Analysis

**Figure 5:**
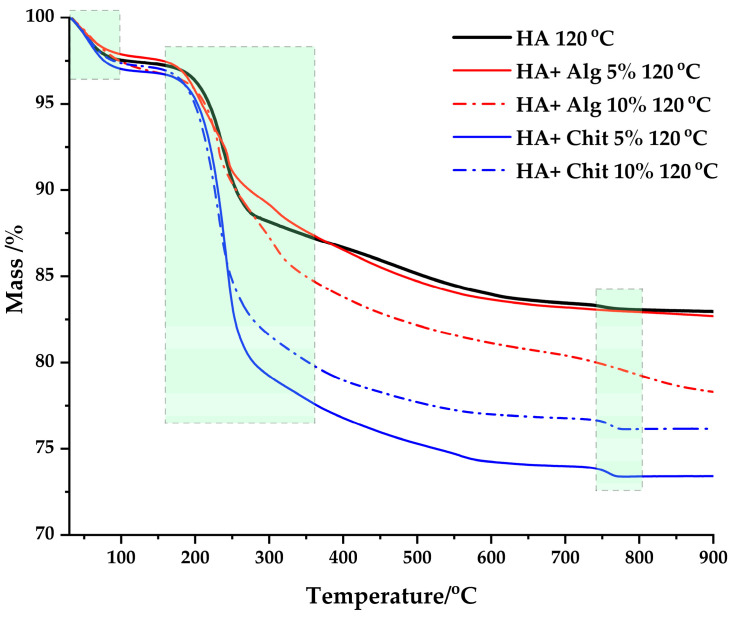
TG curves of samples dried at 120 °C.

**Figure 6:**
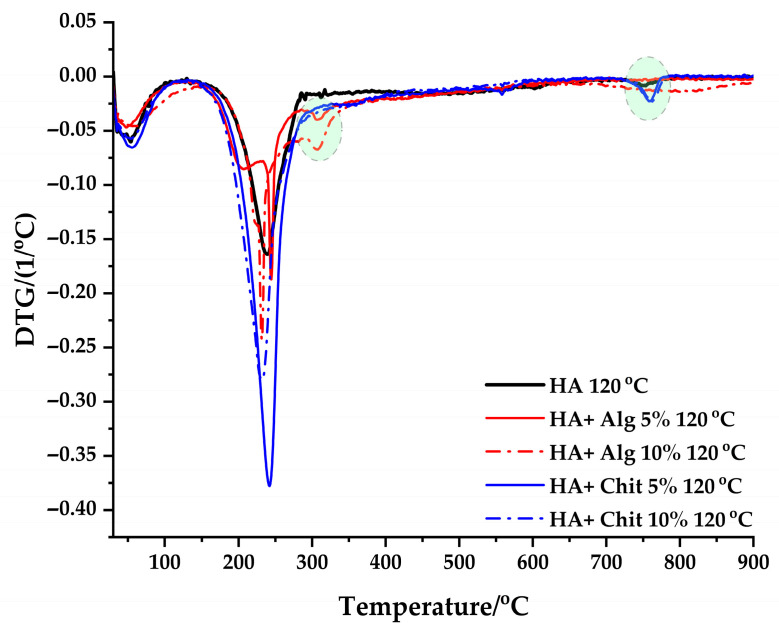
DTG curves of samples dried at 120 °C.

Thermogravimetric analysis (TG) is performed in the temperature range 30–900 °C in nitrogen atmosphere for samples dried at 120 °C. Figure 5 represents the mass loss of the samples analyzed in the chosen temperature range. In addition, in Figure 6 (first order derivative) are distinguished the processes that occur during the heat treatment.

According to the thermal behaviour of the samples, a mass loss of 13% is observed in the case of the HA 120 °C sample and 27% in the case of the HA + Chit 5% 120 °C sample, mass losses that represent the minimum loss (HA 120 °C) and the maximum loss (HA + Chit 5% 120 °C) observed for the analyzed samples. The TG curves are similar to the curve of the HA 120 °C sample, the differences in % mass loss being caused by the added mass of polymer.

In the case of samples containing chitosan, the mass loss occurs in two different stages. The first stage is characterized by a peak with a mass loss of 3% in the temperature range 50–110 °C and is associated with the loss of physically adsorbed water molecules. The second peak is associated with the degradation of the polymer chains and the products resulting from the degradation. The second mass loss process occurs in the temperature range 240–390 °C and presents a mass loss of 21% for HA + Chit 5% 120 °C and 19% for HA + Chit 10% 120 °C, with a maximum mass loss at 230–240 °C [36,37]. From Figure 6, it is observed that the maximum of the second decomposition process decreases from 240 °C to 230 °C, with the increasing amount of chitosan in the respective samples.

For the samples containing alginate, a first process is observed that occurs in the temperature range 50–110 °C and is associated with the loss of physically adsorbed water, being characterized by a process with a mass loss of 2%. The second stage occurs in the range of 180–300 °C and is associated with a process of decomposition of the alginate carbon chains, having a mass loss of 9% for HA + Alg 5% 120 °C and 11% for HA + Alg 10% 120 °C. From the DTG curves of samples containing alginate, 2 additional processes are distinguished, which occur in the range of 150–210 °C, being associated with the release of water captured in the pores formed following the rearrangement of the polymer chain in the presence of Ca^2+^ ions and the decomposition of the polymer chain. The DTG curves indicate that crosslinking with Ca^2+^ cations influences the degradation temperature value of alginate. It is observed that the degradation stage ends at higher temperatures, approximately 350 °C, compared to samples that do not contain alginate.

In the temperature range 750–800 °C, for the samples HA 120 °C, HA + Chit 5% 120 °C, and HA + Chit 10% 120 °C, a process is observed with a mass loss of approximately 1%. This process is attributed to the decomposition of calcite or the phase transformation of HA into β-TCP [38].

Thermogravimetric analysis correlates with observations made from FT-IR and XRD analyses as follows:

- Indicates the formation of crystalline hydroxyapatite in samples containing alginate.
- Presence of impurities or formation of compounds other than those expected following heat treatment.

#### 3.1.4. EDAX Analysis

Following EDAX analysis, the Ca and P content of the analyzed samples and the quantitative changes that occur after heat treatment are monitored. Thus, the possible substance present in the analyzed sample can be identified, depending on the Ca/P ratio.

In Table 3 and Table 4, the percentage content of Ca and P for the samples dried at 120 and 1000 °C determined experimentally is found, and the spectra of the EDAX analysis obtained from which the Ca/P ratio was determined are represented in Appendix A by Figure A6, Figure A7, Figure A8, Figure A9, Figure A10, Figure A11, Figure A12, Figure A13, Figure A14 and Figure A15.

Since the Ca/P ratio of stoichiometric hydroxyapatite is equal to 1.667, it is considered acceptable for this ratio to be situated between 1.658 (corresponding to an HA powder containing 5% β-TCP) and 1.824 (HA + 5% CaO powder) [39]. For samples dried at 120 °C, it is observed that only the HA and HA + Chit 5% samples are within the range reported in the literature, and the HA + Alg 10% and HA + Chit 10% samples exceed this range relatively little.

The ratios resulting from calcination show a decrease in the Ca/P ratio for all samples. In the case of the HA + Alg 5% sample, we have the closest ratio value of 1.667, which indicates the obtaining of a purer hydroxyapatite. Also, the HA + Chit 10% sample pointed out, which has a Ca/P ratio of 1.577, a ratio close to the Ca/P ratio present in β-TCP of 1.5.

The results obtained in each analysis are consistent with each other and provide information on the evolution of the samples during calcination. It is known that increasing the temperature results in a higher crystallinity of the samples. In fact, the HA formation reaction comprises several successive steps: first, the formation of octacalcium phosphate (OCP), which is rapidly transformed into amorphous calcium phosphate (ACP), which is then transformed into HA [40]. FT-IR spectroscopy and thermogravimetric analysis provide information on the synthesized compounds and the possibility of the presence of other substances than the desired ones as impurities in the analyzed samples. XRD analysis successfully identified the phases present in the samples, and EDAX analysis provided additions to those observed.

### 3.2. Evaluation of Dimensional Parameters

Using the available analytical methods (XRD, DLS, and SEM), the data obtained for samples dried at 120 °C and calcined at 1000 °C were consolidated to observe changes in particle size resulting from the heat treatment.

#### 3.2.1. DLS Analysis

From the DLS analysis, the average particle diameter was centralized in Table 5.

A decrease in particle diameter was observed in all samples. This was most evident in the HA + Alg 10% sample, where a 67.7% decrease in particle diameter was seen after heat treatment. In the case of the HA sample, a decrease in particle diameter of 12.83% was noted. For the chitosan samples, a decrease in particle diameter of 16.53% (HA + Chit 5%) and 17.42% (HA + Chit 10%) was observed. A decrease of 46.47% was observed in the HA + Alg 5% sample.

Following this analysis, it was found that the addition of polymer at lower concentrations (5%) at the time of reaction leads to a reduction in particle diameter of up to 50% for the samples dried at 120 °C, and in the case of the addition of the polymer solution at a concentration of 10%, a decrease in particle size of 17.7% was obtained. The heat treatment leads to a reduction in particle diameter for all samples, with alginate bringing about the most significant change.

#### 3.2.2. XRD Analysis

The XRD analysis provided details of the phases present in each sample before and after heat treatment, as well as details of the crystal size of the identified phases. These details are summarized in Table 6 for the predominant phases identified in the samples.

Based on the parameters obtained after XRD analysis for the samples dried at 120 °C, it can be observed that the size of the crystals obtained after synthesis is similar. Except for the HA + Alg 10% sample, in which the crystal size is larger than in the other samples.

By applying the heat treatment, the phases present in the samples change their dimensional parameters, and the crystal size increases significantly. The crystal size thus increases with temperature, but, since high temperatures favour nucleation, the particles tend to agglomerate with each other, and larger average sizes of the agglomerates are observed [41].

#### 3.2.3. SEM Analysis

**Figure 7:**
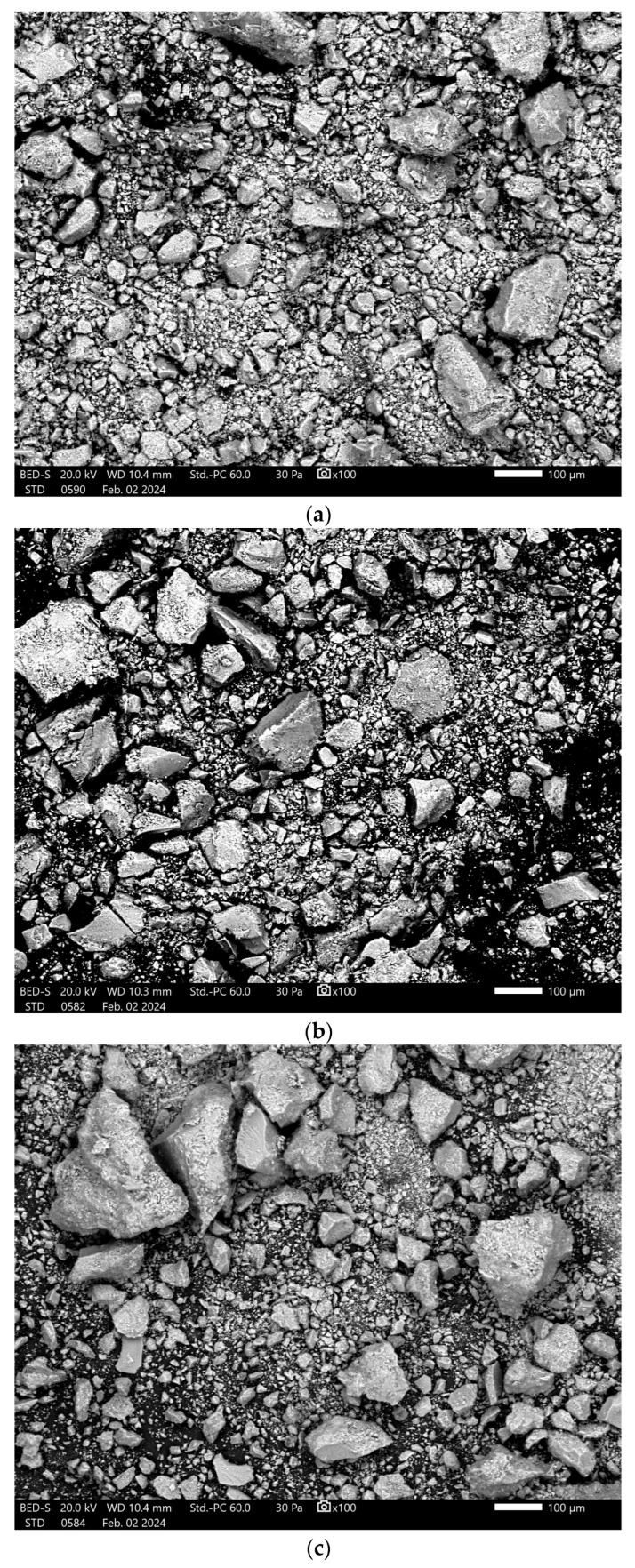

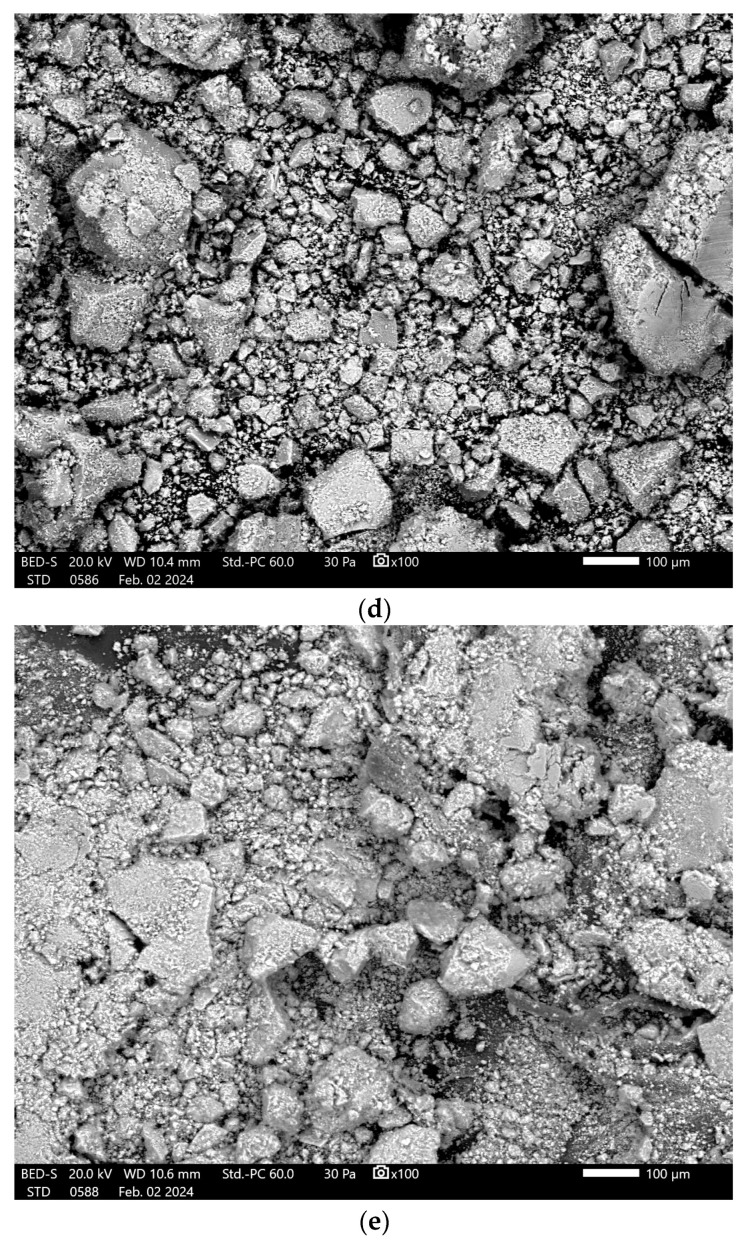
SEM images at ×100 magnification for the samples (**a**) HA 120 °C; (**b**) HA + Alg 5% 120 °C; (**c**) HA + Alg 10% 120 °C; (**d**) HA + Chit 5% 120 °C and; (**e**) HA + Chit 10% 120 °C.

The SEM images of the samples provide information about the shape and distribution of the particles in the analyzed samples. Figure 7 shows the analyzed powders for the samples dried at 120 °C.

The presence of agglomerates with sizes between 100 and 150 µm is evident in all samples. In addition, smaller particles between 10 and 25 µm are distinguished, but a clearly defined shape is not observed for the analyzed samples. The largest sizes were identified in the HA + Chit 10% sample.

**Figure 8:**
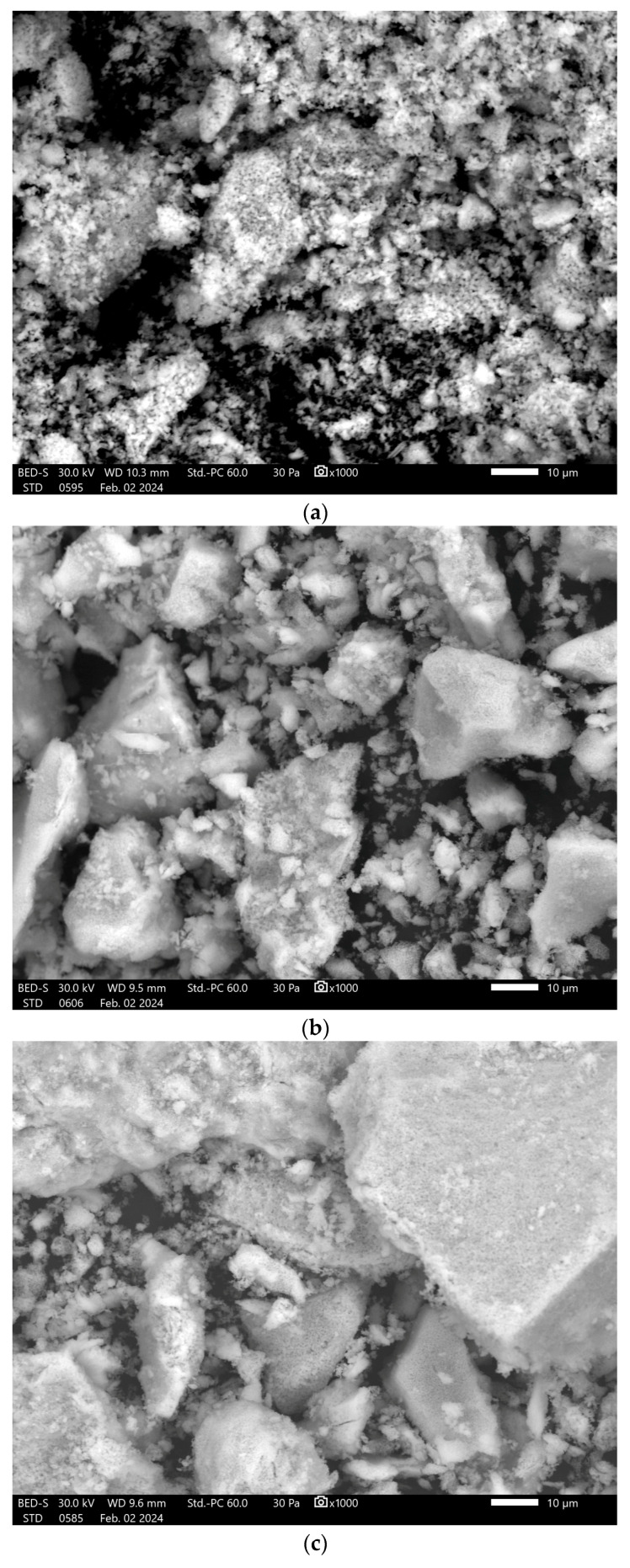

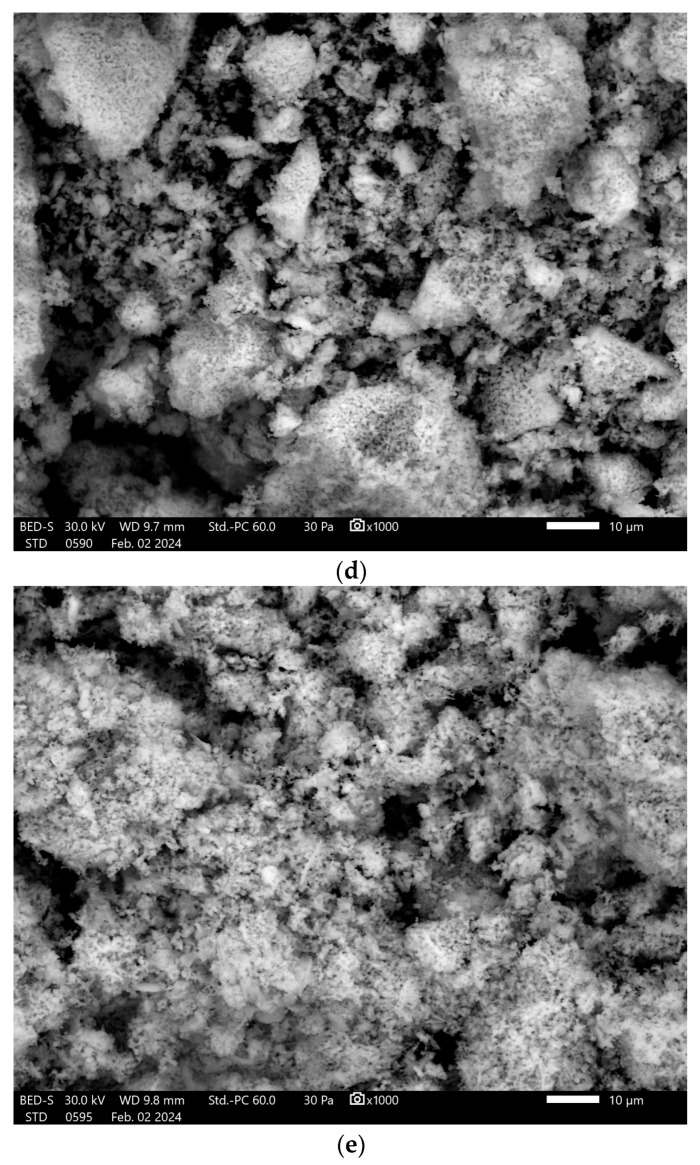
SEM images at ×1000 magnification for the samples (**a**) HA 1000 °C; (**b**) HA + Alg 5% 1000 °C; (**c**) HA + Alg 10% 1000 °C; (**d**) HA + Chit 5% 1000 °C and; (**e**) HA + Chit 10% 1000 °C.

**Figure 9:**
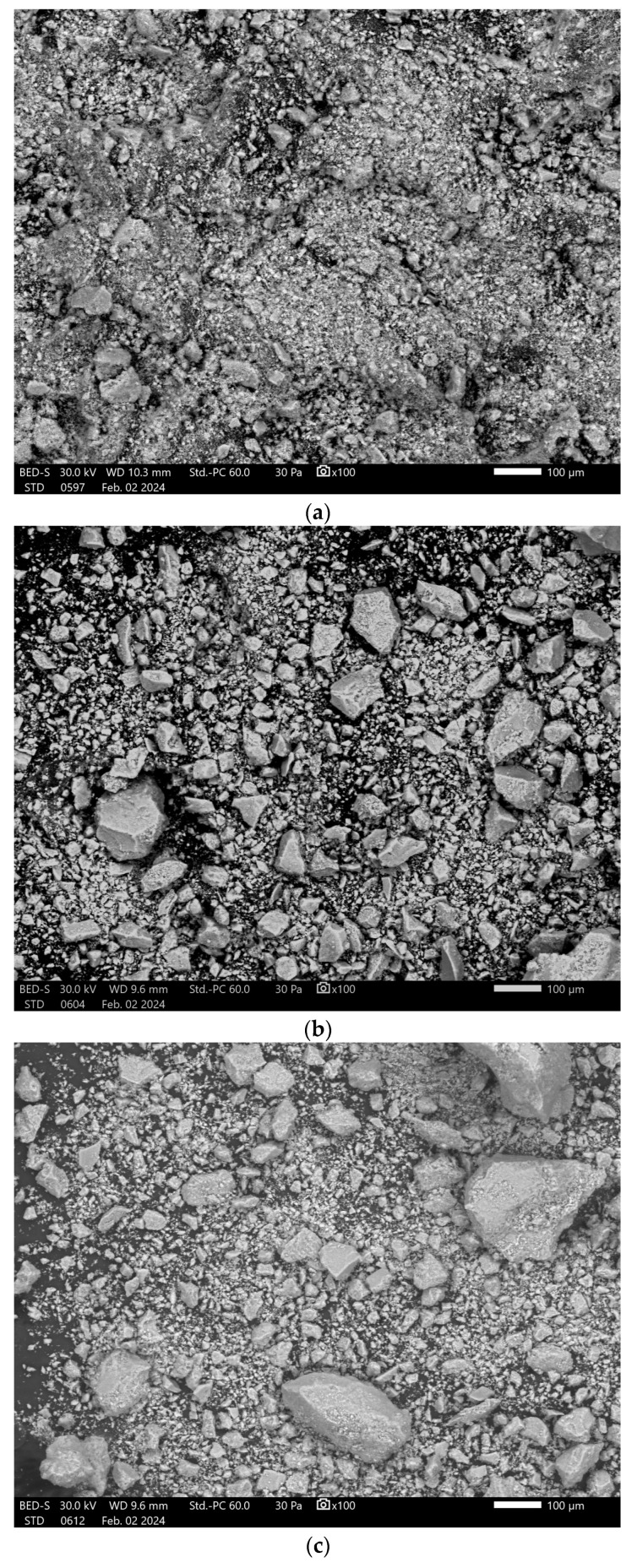

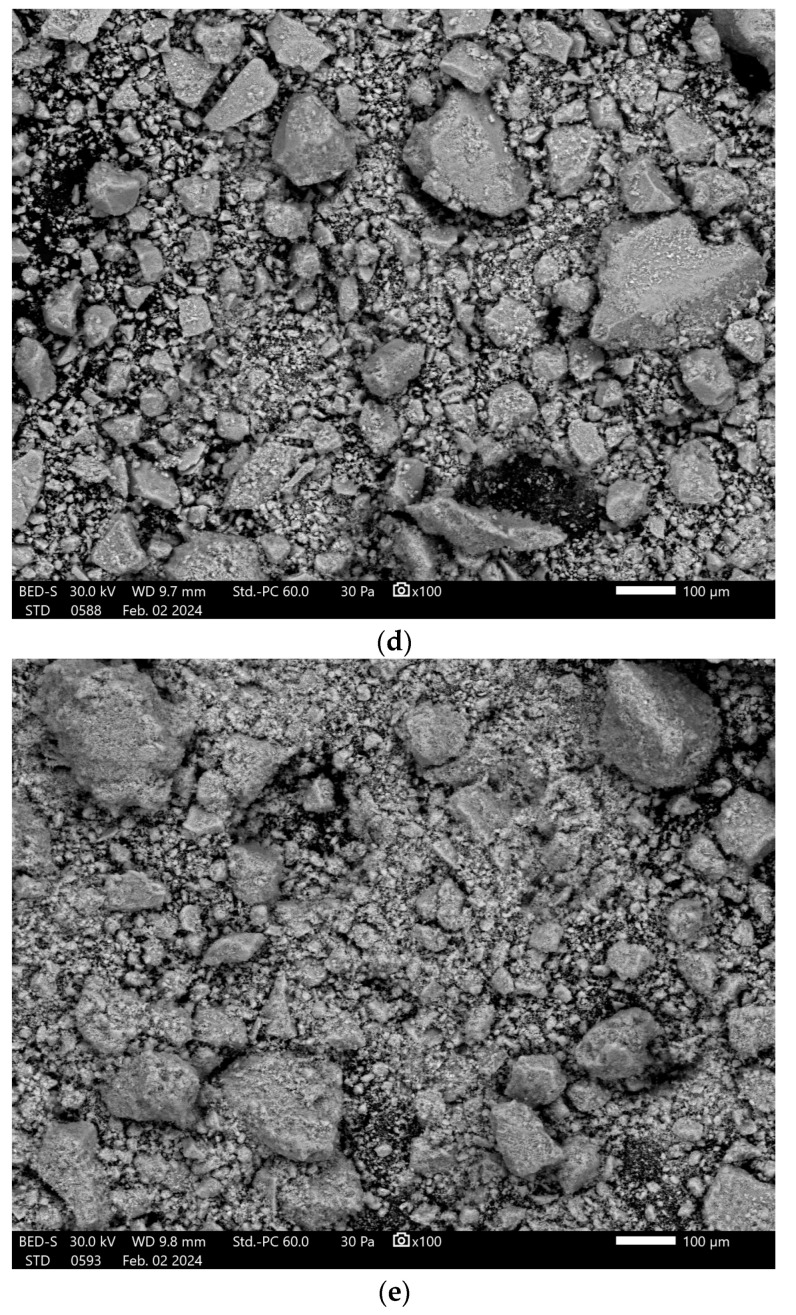
SEM images at ×100 magnification for the samples (**a**) HA 1000 °C; (**b**) HA + Alg 5% 1000 °C; (**c**) HA + Alg 10% 1000 °C; (**d**) HA + Chit 5% 1000 °C and; (**e**) HA + Chit 10% 1000 °C.

After heat treatment, the morphology of the samples changes and the agglomerates largely disappear. At a magnification of 1000× (Figure 8), well-defined pores can be observed on the particle surface, with dimensions smaller than 1 µm. SEM images of samples heat-treated at 1000 °C show a decrease in the size of the particles and agglomerates present in the sample at a magnification of 100× (Figure 9).

The XRD data indicates an increase in crystallite size after calcination by more than ten times, while the DLS and SEM analyses show a decrease in agglomerate size. This discrepancy is caused by the fact that larger crystallites generally have a lower tendency to agglomerate compared to smaller ones [42,43,44].

## 4. Conclusions

The characterization of the samples provides valuable information about the synthesis and how the presence of polymers influences the synthesis result. By directly comparing the use of an anionic and a cationic polymer under similar synthesis conditions, it was shown that the use of the anionic polymer, alginate, in the concentration range studied lowers the particle size of hydroxyapatite the most.

The FT-IR analysis shows the complete disappearance of the characteristic bands of the polymers after the application of the heat treatment at the selected temperature. It also shows the presence of some secondary compounds of the synthesis compared to the desired reaction. After calcination, the presence of the characteristic bands of the hydroxyl group at 3373 and 663 cm^−1^ and the phosphate group in the HA and alginate-containing samples indicates the presence of hydroxyapatite in these samples. In contrast, for the samples containing chitosan, the appearance of bands characteristic of β-tricalcium phosphate and hydrogen phosphate was observed.

The XRD analysis confirmed the presence of possible phases identified by FT-IR spectroscopy. In addition to the phases identified in the FT-IR spectra, the presence of calcite and tetracalcium phosphate was identified in relatively small proportions. Thermogravimetric analysis and EDAX analysis confirmed the FT-IR and XRD results.

The change in dimensional parameters is monitored using DLS, XRD, and SEM analyses. The DLS analysis shows a decrease in particle size after heat treatment. The largest decrease in the average particle diameter is 67.7% for the HA + Alg 10% sample, but a decrease in particle diameter is observed for all samples. This is more evident in the SEM analysis, where a decrease in the presence of agglomerates and the size of smaller particles can be observed after heat treatment.

In this case, the XRD analysis shows an increase in crystal size, independent of the identified phase. This is in accordance with data from the literature, where it has been shown that thermal treatment of hydroxyapatite precursors leads to an increase in crystal size due to the merge of smaller crystallites and the formation of crystalline hydroxyapatite.

This study has thus demonstrated the possibility of using polymers to lower the particle size of hydroxyapatite, with the alginate giving better results due to the negative charge of the polymer chain in solution and its affinity for divalent cations.

In addition, the heat treatment leads to phosphate compounds that exhibit increased porosity and may have applicability in the field of tissue engineering after further investigations, such as bioactivity and cytocompatibility.

The concentration range of the alginate solution should be modified in future work to both lower and higher concentrations to reveal how the viscosity of the polymer solution can affect the properties of the resulting particles.

## Figures and Tables

**Table 1:** Summary of the samples and their abbreviations.

Set I (Dried at 120 °C)	Set II (Calcined at 1000 °C)
HA	HA
HA + Alg 5%	HA + Alg 5%
HA + Alg 10%	HA + Alg 10%
HA + Chit 5%	HA + Chit 5%
HA + Chit 10%	HA + Chit 10%

**Table 2:** Percentage composition of samples calcined at 1000 °C.

Sample 1000 °C	Mass Fraction of the Identified Phase (%)			
	HA	β–TCP	CaCO_3_	Ca_4_(PO_4_)_2_O
HA	54.96	40.16	0.99	3.89
HA + Alg 5%	95.85	3.49	0.66	-
HA + Alg 10%	88.3	2.22	2.03	3.90
HA + Chit 5%	-	95.34	-	4.66
HA + Chit 10%	-	95.12	0.99	3.89

**Table 3:** Percentage composition of samples dried at 120 °C.

Sample 120 °C	Ca (%)	P (%)	Ca/P Ratio
HA	32.07 ± 0.21	17.93 ± 0.13	1.788
HA + Alg 5%	33.14 ± 0.19	17.39 ± 0.12	1.905
HA + Alg 10%	28.43 ± 0.16	15.35 ± 0.10	1.852
HA + Chit 5%	34.46 ± 0.20	19.14 ± 0.13	1.797
HA + Chit 10%	34.87 ± 0.24	18.82 ± 0.15	1.852

**Table 4:** Results obtained from EDAX analysis for samples calcinated at 1000 °C.

Sample 1000 °C	Ca (%)	P (%)	Ca/P Ratio
HA	36.65 ± 0.16	21.07 ± 0.12	1.739
HA + Alg 5%	19.45 ± 0.07	11.53 ± 0.05	1.686
HA + Alg 10%	22.70 ± 0.08	12.90 ± 0.06	1.759
HA + Chit 5%	28.80 ± 0.11	16.74 ± 0.08	1.720
HA + Chit 10%	23.98 ± 0.09	15.20 ± 0.07	1.577

**Table 5:** The average diameter of the particles in the analyzed samples.

	Average Diameter/nm	
Sample	120 °C	1000 °C
HA	2649	2309
HA + Alg 5%	1360	728
HA + Alg 10%	2180	704
HA + Chit 5%	1421	1186
HA + Chit 10%	2181	1801

**Table 6:** Identified phases and crystal size in the studied samples.

	Identified Phase/Temperature			
Sample	Phase	120 °C	Phase	1000 °C
HA	HA	(0,0,1) = 8.98 ± 0.39 nm (1,0,0) = 8.49 ± 0.29 nm	β-TCP	(1,1,1) = 149.8 ± 1.7 nm
			HA	(0,0,1) = 116.8 ± 5.8 nm (1,0,0) = 121.2 ± 3.6 nm
HA + Alg 5%	HA	(0,0,1) = 7.87 ± 0.68 nm (1,0,0) = 9.09 ± 0.50 nm	HAm	A = 0.9417 nm B = 1.8848 nm C = 0.6888 nm
HA + Alg 10%	HA	(0,0,1) = 26.83 ± 0.58 nm (1,0,0) = 14.23 ± 0.16 nm	HA	(1,1,1) = 104 ± 15 nm
HA + Chit 5%	HA	(0,0,1) = 9.57 ± 0.40 nm (1,0,0) = 8.49 ± 0.21 nm	β-TCP	(1,1,1) = 161.7 ± 1.7 nm
HA + Chit 10%	HA	(0,0,1) = 8.49 ± 0.28 nm (1,0,0) = 8.49 ± 0.15 nm	β-TCP	(1,1,1) = 128.1 ± 1.2 nm

**Figure A6:**
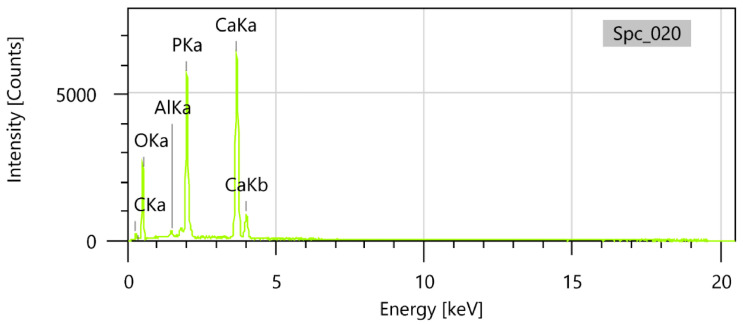
EDAX analysis spectra for the sample HA 120 °C.

**Figure A7:**
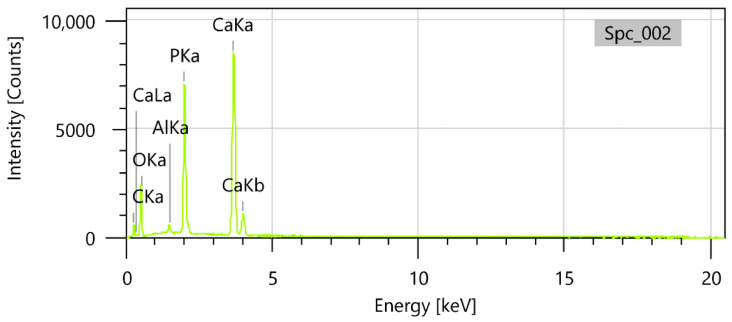
EDAX analysis spectra for the sample HA + Alg 5% 120 °C.

**Figure A8:**
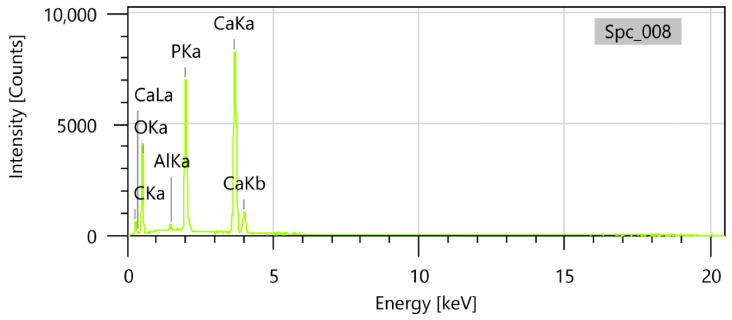
EDAX analysis spectra for the sample HA + Alg 10% 120 °C.

**Figure A9:**
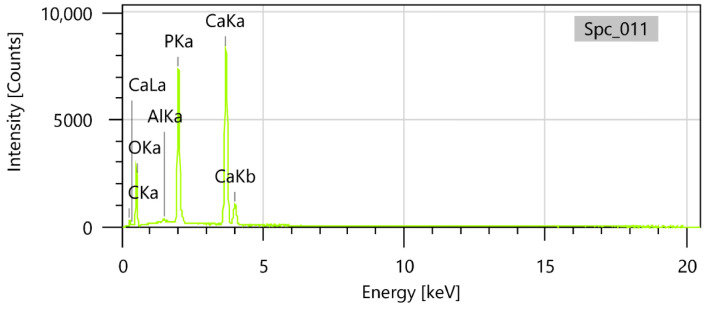
EDAX analysis spectra for the sample HA + Chit 5% 120 °C.

**Figure A10:**
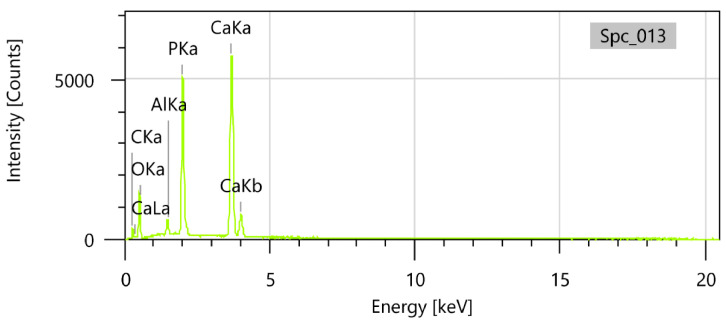
EDAX analysis spectra for the sample HA+ Chit 10% 120 °C.

**Figure A11:**
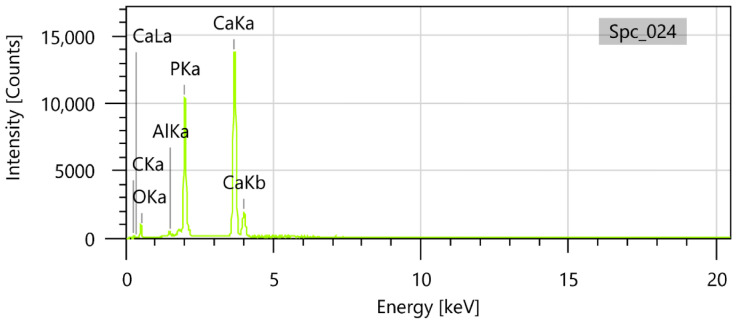
EDAX analysis spectra for the sample HA 1000 °C.

**Figure A12:**
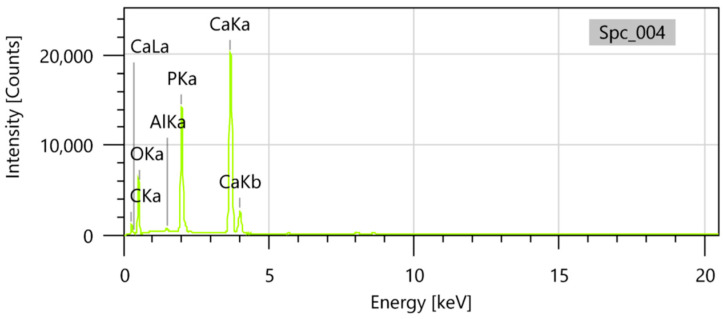
EDAX analysis spectra for the sample HA + Alg 5% 1000 °C.

**Figure A13:**
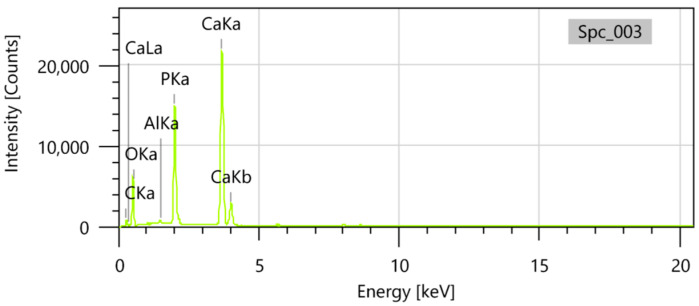
EDAX analysis spectra for the sample HA + Alg 10% 1000 °C.

**Figure A14:**
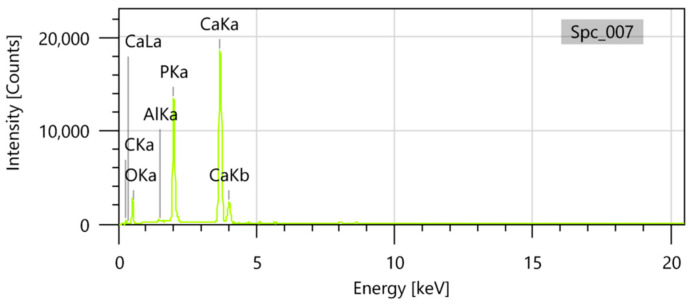
EDAX analysis spectra for the sample HA + Chit 5% 1000 °C.

**Figure A15:**
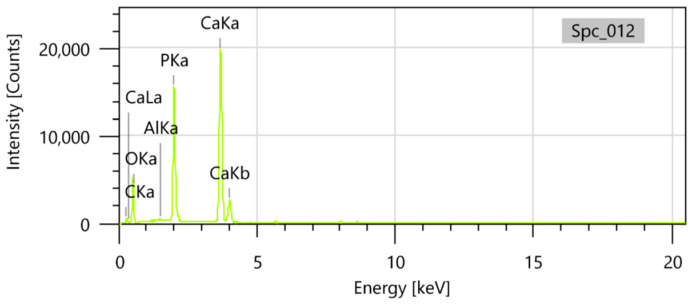
EDAX analysis spectra for the sample HA + Chit 10% 1000 °C.

## Data Availability

The data presented in this study are available on request from the corresponding author.
